# Duodenal Small Bowel Diaphragm Disease: A Rare and Underrecognized Complication of Chronic NSAID Use

**DOI:** 10.14309/crj.0000000000002026

**Published:** 2026-02-20

**Authors:** Idan Grossmann, Aubin Attila, Harshavardhan Sanekommu, Chinmay Trivedi, Vera Hapshy, Natasha Campbell, Lee Peng, Angelo Chinnici, Mohammad Hossain

**Affiliations:** 1 Department of Internal Medicine, Jersey Shore University Medical Center, Neptune, NJ; 2 Division of Gastroenterology, Jersey Shore University Medical Center, Neptune, NJ; 3 Division of Gastroenterology, Hackensack Meridian Palisades Medical Center, North Bergen, NJ

**Keywords:** nonsteroidal anti-inflammatory drugs (NSAIDs), small bowel diaphragm disease, duodenal obstruction, small bowel pathology, small bowel obstruction

## Abstract

Nonsteroidal anti-inflammatory drugs (NSAIDs) are among the most commonly used medications. Although common adverse effects such as gastrointestinal bleeding, ulcers, and kidney injury are widely known, small bowel diaphragm disease, an exceedingly rare complication of long-term NSAID use, remains underrecognized. We report an uncommon case of a 56-year-old patient on long-term high-dose NSAIDs who developed diaphragm disease involving the duodenum, a rarely reported location, manifesting as intestinal obstruction. This case highlights the potential for unusual anatomical involvement to mask diagnosis and underscores the importance of early consideration of this diagnosis in patients with chronic NSAID use and unexplained obstructive presentations.

## INTRODUCTION

Nonsteroidal anti-inflammatory drugs (NSAIDs) are widely used worldwide. Although their well-known side effects, such as gastritis and peptic ulcer disease, are recognized, a rare but clinically significant complication, small bowel diaphragm disease, remains underappreciated.

NSAIDs decrease the production of protective prostaglandins, which results in small bowel damage. Prostaglandin-mediated protection reduces mucosal blood flow and impairs epithelial healing, predisposing the small bowel to repeated cycles of ulceration and fibrosis. Recurrent mucosal injury leads to ulceration and subsequent healing with fibrosis, forming strictures that narrow the intestinal lumen and may result in obstruction.^1,2^

Due to its rarity and nonspecific presentation, small bowel diaphragm disease often remains undiagnosed until complications arise. Diagnosis typically requires a high index of suspicion and is often confirmed only through invasive intervention such as esophagogastroduodenoscopy (EGD) and surgical exploration.

This report highlights a rare case of duodenal diaphragm disease in a patient on long-term high-dose NSAIDs, emphasizing the importance of considering this adverse effect when prescribing NSAIDs and when evaluating unexplained gastrointestinal obstruction in high-risk patients.

## CASE REPORT

A 56-year-old woman with a history of myasthenia gravis and chronic pain syndrome managed with high-dose ibuprofen (800 mg every 6 hours for 5 years) after a motor vehicle collision presented with a 1-month history of severe epigastric pain, accompanied by nausea, bloating, and poor appetite. She denied fever, vomiting, hematemesis, melena, hematochezia, history of inflammatory bowel disease, colon cancer, prior abdominal surgery, or radiation. Physical examination revealed generalized abdominal tenderness with distention and hypoactive bowel sounds but no peritoneal signs.

Laboratory findings showed leukocytosis with a white blood cell count of 13.3 × 10^9^/L with 84% neutrophilia, and serum lactic acid was within normal limits. Abdominal X-ray demonstrated a markedly dilated air-filled stomach with air-fluid levels, raising concern for gastric outlet obstruction.

CT imaging was obtained to localize the site and nature of obstruction; it showed significant dilation of the stomach and proximal duodenum with a focal transition point and segmental thickening in the third portion of the duodenum, suggesting an intrinsic obstructing lesion.

EGD was performed to assess intraluminal causes and revealed a severely distended stomach with a stricture in the third portion of the duodenum. Push enteroscopy was pursued for further visualization. Endoscopic dilation was not attempted due to the fixed, circumferential fibrosis and high perforation risk. Persistent, fixed obstruction despite endoscopic evaluation necessitated exploratory laparotomy, which demonstrated severe inflammation and edema of the proximal duodenum requiring segmental resection.

Histopathologic examination demonstrated severe serosal fibrosis with dense adhesions. At the site of narrowing, the mucosa showed ulceration, granulation tissue, and marked congestion. The submucosa exhibited concentric fibrotic thickening extending into the muscularis propria, forming a characteristic diaphragm-like circumferential stricture.

Other causes of duodenal obstruction were excluded: there was no ulcer scarring on endoscopy; no mass or lymphadenopathy to suggest malignancy; and no clinical, imaging, or histologic features of Crohn's disease, ischemia, tuberculosis, or neoplasm. Given these findings, the patient's long-term NSAID use, and the characteristic histologic features, the diagnosis was consistent with NSAID-induced small bowel diaphragm disease.

NSAIDs were discontinued, and the patient received IV proton-pump inhibitors, nasogastric decompression, and bowel rest. After surgical resection, her symptoms resolved, and she was discharged with outpatient follow-up.

## DISCUSSION

Small bowel diaphragm disease is a complication of long-standing NSAID use, first described in 1988.^3^ Fewer than 100 cases have been reported (Table 1). Most involve the ileum or jejunum, whereas duodenal involvement is exceptionally rare. The limited cases reflect variable presentations and challenges in timely diagnosis, requiring high suspicion.

**Table 1.** Reported cases of NSAID-associated small bowel diaphragm disease

Author	Patients	NSAID use,	Location	Presentation	Diagnosis	Management
Lang et al^2^	8	3–7	Ileum, jejunum	Obstruction, anemia	Surgery/pathology	Resection
Flicek et al^4^	12	5–10	Ileum, jejunum	Obstruction, anemia	CT, surgery	Surgery
Sessler et al^5^	55	7, avg	Ileum > jejunum, duodenum rare	Obstruction, anemia	Surgery, capsule	Surgery 51%, capsule 25%
Kelly et al^6^	7	≥5	Jejunum, ileum	Obstruction, pain	Surgery	Resection
Al-Feghali et al^1^	1	5	Ileum	Intussusception	CT, surgery	Resection
Jeong et al^7^	1	6	Small bowel (cluster)	Obstruction	CT, endoscopy	Surgery
Ballal et al^8^	1	4–6	Jejunum, ileum	Pain, obstruction	Endoscopy, surgery	Resection
Pereira and Slater^9^	1	5	Ileum	Obstruction, pain	Endoscopy, surgery	Surgery
Johnson et al^10^	1	6	Ileum	Intestinal stricturing	CT, surgery	Resection
Karam et al^3^	1	4	Terminal ileum	Obstruction, pain	Endoscopy	Surgery

CT, computed tomography; NSAID, nonsteroidal anti-inflammatory drug.

The clinical presentation is often nonspecific and can range from being asymptomatic to presenting with symptoms of bowel obstruction. Because of its rarity and the symptoms it presents with, small bowel diaphragm disease, an uncommon cause of small intestinal obstruction, is frequently overlooked.^7–10^

NSAIDs inhibit prostaglandin production, reducing mucosal blood flow and impairing healing, which leads to repeated ulceration and fibrosis, ultimately causing strictures that may obstruct the intestinal lumen.^2^

A retrospective study of 12 patients with pathologically proven small-bowel diaphragm disease found abdominal pain and anemia as the most common presentations, with strictures primarily in the ileum.^4^ Sessler et al^5^ reviewed 55 surgically confirmed cases, 44 of which were associated with NSAID use. The average exposure duration was 7 years, with ileal involvement predominating and duodenal involvement in fewer than 5%, highlighting the rarity of our case. Similarly, Ballal et al^8^ reported diaphragm disease affecting the jejunum and ileum, which are more commonly involved. Kelly et al^6^ described scattered diaphragms throughout the ileum and jejunum in 7 patients, all requiring surgical resection.

Only a handful of duodenal cases of NSAID-induced diaphragm disease have been reported, with Wang et al^11^ identifying 3 duodenal cases in their 159-patient review, making our presentation of an isolated obstructing diaphragm in the third portion of the duodenum particularly uncommon.

**Figure 1.**
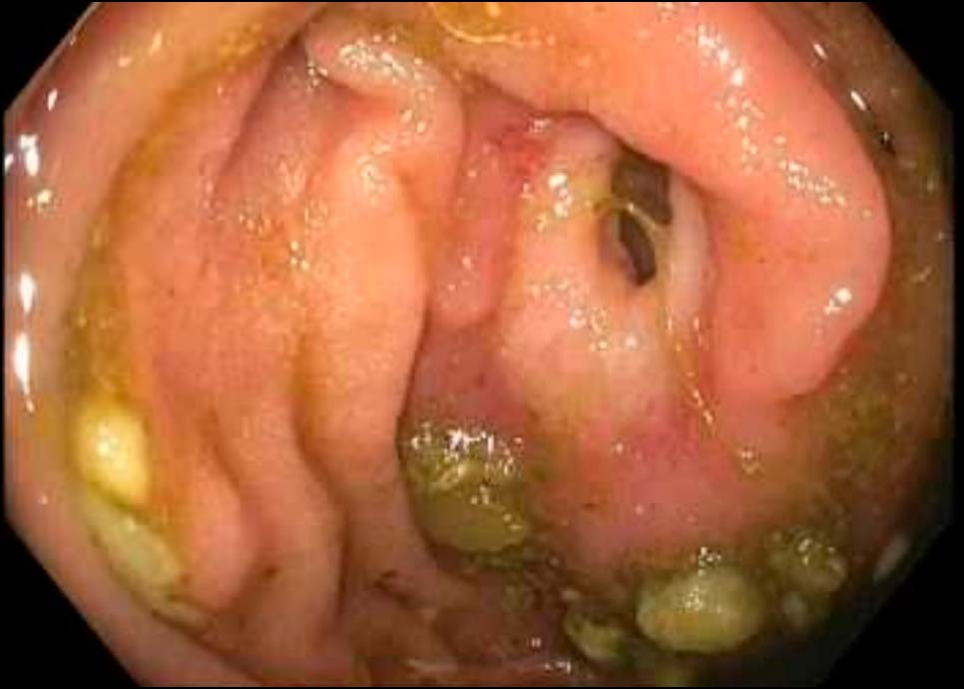
A stricture of the third portion of the duodenum.

**Figure 2.**
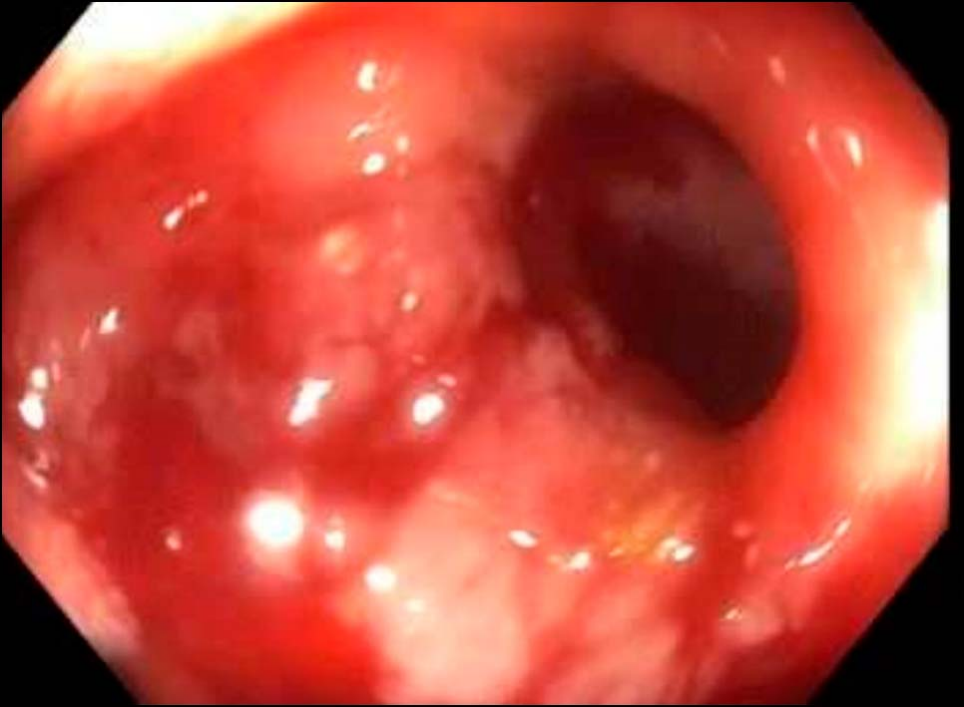
Erythematous and edematous mucosa of the duodenal wall.

Our patient's disease showed uncommon involvement of the duodenum with obstructive features, a presentation reported in a limited number of prior cases,^1^ representing a highly unusual site of involvement and underscoring the critical importance of recognizing diaphragm disease in atypical presentations (Figures 1 and 2).

Diagnosis and management of small bowel diaphragm disease are challenging due to nonspecific symptoms and often require invasive evaluation. Our patient, with long-term NSAID use, needed EGD, push enteroscopy, and surgical resection. Review of 55 cases showed laparotomy in 51% and capsule endoscopy in 25%, highlighting the need for suspicion and early intervention, especially with atypical duodenal involvement.^5^

This case highlights the importance of recognizing NSAID-induced small bowel diaphragm disease in the differential diagnosis of unexplained gastrointestinal symptoms, especially in chronic NSAID users. Atypical duodenal involvement, as in our patient, can obscure diagnosis by mimicking other conditions. Early identification is critical, as timely evaluation and intervention prevent delays and improve outcomes.

Clinical pearl: Suspect diaphragm disease in chronic NSAID users with unexplained obstruction; use CT/MR enterography first, and escalate to device-assisted enteroscopy or surgery if imaging does not provide a clear diagnosis.

## DISCLOSURES

Author contributions: I. Grossmann: Conceptualization of the case report, drafting of the manuscript, data collection, and final approval of the submitted version. A. Attila: Manuscript review and editing. H. Sanekommu: Gastroenterology consultation, interpretation of endoscopic findings, and manuscript review. C. Trivedi: Gastroenterology consultation, interpretation of endoscopic findings, and manuscript review. V. Hapshy: Literature review and manuscript editing. N. Campbell: Manuscript review and editing. L. Peng: Gastroenterology oversight, critical revisions related to clinical interpretation, and final manuscript approval. A. Chinnici: Manuscript review and final manuscript approval. M. Hossain: Manuscript review and final manuscript approval. M. Hossain is the article guarantor and will serve as the article guarantor and accepts full responsibility for the integrity of the work, including the conduct of the study, the accuracy of the data, and the final manuscript content.

Financial disclosure: None to report.

Informed consent was obtained for this case report.

## ABBREVIATIONS:

CT: Computed Tomography
CT/MR: Computed Tomography/Magnetic Resonance Imaging
EGD: Esophagogastroduodenoscopy
IV: Intravenous
NSAID: Nonsteroidal Anti-Inflammatory Drug
